# Latent Autoimmune Diabetes in Adults: A Case Series and Clinical Insights

**DOI:** 10.7759/cureus.90879

**Published:** 2025-08-24

**Authors:** Trupti Pisal, Preeti R Gupta, Dhanashree B Mestry, Avinash D Arke

**Affiliations:** 1 Internal Medicine, Jagjivanram Railway Hospital, Mumbai, IND; 2 Cardiology, Jagjivanram Railway Hospital, Mumbai, IND

**Keywords:** anti-glutamic acid decarboxylase antibodies, autoimmune, c-peptide level, diabetes mellitus, islet beta-cell function, latent autoimmune diabetes in adults

## Abstract

Introduction: Diabetes mellitus (DM) is a disease spectrum ranging from classic insulin deficiency of type 1 diabetes (T1DM) to insulin-resistant type 2 diabetes (T2DM). Latent autoimmune diabetes in adults (LADA) is a type of DM with clinical characteristics of both T1DM and T2DM. Despite its clinical significance, LADA remains underdiagnosed and mismanaged due to overlapping characteristics with other diabetes types.

Methods: It is a case series describing clinical, immunological, and metabolic characteristics of ten patients diagnosed with LADA based on three criteria identified by the Immunology of Diabetes Society.

Results: We found that these patients had uncontrolled diabetes not controlled with oral hypoglycemic drugs, were mostly males aged above 30 years with a mean body mass index of 22.6 kg/m², and everyone had positive anti-glutamic acid decarboxylase (GAD) antibody. At the presentation, one patient had combined moderate non-proliferative diabetic retinopathy and peripheral neuropathy. Four patients had mild non-proliferative diabetic retinopathy, and two had peripheral neuropathy as complications of uncontrolled diabetes.

Conclusion: LADA remains a diagnostic challenge, as its phenotypic picture resembles T2DM, while immunologically it resembles T1DM. A high index of suspicion, early diagnosis, and correct treatment can prevent further damage to the beta islet cells and help prevent short-term and long-term complications of DM.

## Introduction

Diabetes is a chronic metabolic disease with serious long-term complications. Elevated blood glucose concentrations may be related to abnormal secretion or action of insulin. Diabetes mellitus (DM) is a global health issue, with an estimated 537 million adults (20-79 years) suffering from DM worldwide in 2021 [1]. In India, 89.8 million adults had diabetes in 2024, and it is estimated to increase to 156.7 million adults by 2050 [2]. Latent autoimmune diabetes in adults (LADA) is a type of diabetes characterized by adult-onset of the disease and circulating autoimmune antibodies; thus, it shares clinical characteristics of both type 1 (T1DM) and type 2 diabetes (T2DM) [3-5]. It accounts for an estimated 2-18% of all adults with diabetes [4,6-8].

LADA often remains underdiagnosed or misdiagnosed as T2DM due to a lack of universally accepted diagnostic criteria, leading to a delay in initiating appropriate therapy, prolonged exposure to the deleterious effects of hyperglycemia, and unwarranted exposure to ineffective oral hypoglycemic agents. The Immunology of Diabetes Society has laid down diagnostic criteria to diagnose LADA [9]. The patient should be ≥ 30 years old; have at least one of the four antibodies commonly seen in T1DM, namely anti-glutamic acid decarboxylase (GAD) autoantibodies, insulin autoantibodies, islet cell antibodies (ICA), and insulinoma-associated-2 autoantibodies; and not be on insulin treatment for the first six months.

There is limited literature on the clinical spectrum of LADA in Indian adults. The aim of this case series is to discuss the clinical, immunological, and metabolic characteristics of patients with LADA and to provide insights that may facilitate earlier diagnosis and optimize therapeutic decision-making for LADA patients.

## Materials and methods

Study design

The study was conducted at the Jagjivanram Railway Hospital, Mumbai. It is a case series of ten patients with LADA describing their clinical, epidemiological, immunological, and metabolic characteristics.

Study population

Ten patients were diagnosed with LADA at a tertiary care hospital in West India between January 2022 and June 2025.

Procedure and data collection

For the purpose of diagnosis, LADA was diagnosed on the basis of the Immunology of Diabetes Society’s criteria [9]. All the patients above 30 years diagnosed as LADA were included in the study. Those patients younger than 30 years, diagnosed with T1DM, T2DM, or gestational DM, were excluded from the study. The data was collected retrospectively from health records.

The variables evaluated in all patients were demographic and clinical data, including age, sex, body mass index (BMI), age at diagnosis, duration of diabetes, presenting symptoms, family history of DM or autoimmunity, immuno-metabolic data like results of auto-antibody testing (GAD), fasting c-peptide levels, glycated hemoglobin (HbA1c), fasting and postprandial blood sugar levels (FBS, PPBS), glycemic control (HbA1c level), initial treatment regimen (oral hypoglycemic agents vs. insulin), insulin initiation, and data related to long-term complications (retinopathy, nephropathy, and peripheral neuropathy).

The quantitative data were represented as their median ± SD. Categorical data was expressed as a percentage.

## Results

Clinical characteristics

The clinical characteristics of the ten LADA patients are tabulated in Table 1. Of the ten patients, there were six males. The median age of diagnosis of DM and the age at presentation to the hospital were 33 ± 6.5 years (range: 31 to 49 years of age) and 39.5 ± 8.2 years (range: 32 to 52 years), respectively. The average duration of the disease is 5.5 ± 4.4 years. The median body mass index (BMI) was 22.6 ± 1.8 kg/m², ranging from 19.6 to 24.2 kg/m², and the waist circumference was 91.5 ± 4.5. Among the cardiovascular risk factors, two of the ten patients (20%) were hypertensive, whereas all had dyslipidemia with hypertriglyceridemia. Out of the 10 patients, two had Hashimoto’s thyroiditis (20%), and one had pernicious anemia and vitiligo (10%), respectively. Five patients (50%) had a strong family history of T2DM.

**Table 1:** Clinical characteristics of ten LADA patients The variables are expressed as median ± standard deviation. DKA: diabetic ketoacidosis; DPP4: dipeptidyl peptidase 4; NPDR: non-proliferative diabetic retinopathy; LADA: latent autoimmune diabetes in adults; DM: diabetes mellitus

Case No	Sex	Age at presentation	Age at diagnosis	BMI (kg/m²)	Waist circumference (in cm)	Family history of diabetes	Duration of DM (in years)	Initial presentation	Hypertension	Fundoscopy	Other autoimmune conditions	Current treatment
1	M	32	31	20.2	93	No	1	Balanoposthitis	No	Normal	No	DPP4 Inhibitors + Insulin (Basal + Bolus)
2	M	52	44	22.4	96	No	8	Scalp folliculitis	No	Mild NPDR	No	Insulin (Basal + Bolus)
3	M	33	31	22.8	94	No	2	DKA	No	Mild NPDR	Vitiligo	Insulin (Basal + Bolus)
4	M	51	39	19.6	90	No	12	Urinary tract infection	Yes	Normal	No	DPP4 inhibitors + Insulin (Basal + Bolus)
5	F	50	49	21.3	85	No	1	Pneumonia	Yes	Normal	Pernicious anaemia	Insulin (Basal + Bolus)
6	F	43	36	24.2	86	Yes	7	DKA	No	Mild NPDR	Hashimoto's thyroiditis	Insulin (Basal + Bolus)
7	M	36	31	22.4	96	Yes	5	Balanoposthitis	No	Mild NPDR	No	DPP 4 inhibitors, Insulin (Basal + Bolus)
8	M	36	30	23.6	98	Yes	6	Pulmonary tuberculosis	No	Normal	Hashimoto's thyroiditis	Insulin (Basal + Bolus)
9	F	48	35	23.8	88	Yes	13	DKA	No	Moderate NPDR	No	Insulin (Basal + Bolus)
10	F	33	31	25.3	90	Yes	2	DKA	No	Normal	No	DPP 4 inhibitors, Insulin (Basal+Bolus)
Median ± SD		39.5 ± 8.2	33 ± 6.5	22.6 ± 1.8	91.5 ± 4.5		5.5 ± 4.4					

Out of the five patients presented to the casualty and emergency department, four (40%) patients presented with diabetic ketoacidosis (DKA), whereas one had pneumonia. Other patients were admitted through the outpatient department (OPD) with balanoposthitis (two patients), urinary tract infection, scalp folliculitis, and pulmonary tuberculosis (one each). At the time of presentation, one patient (10%) had a combination of moderate non-proliferative diabetic retinopathy and peripheral neuropathy. In addition, four patients (40%) had mild non-proliferative diabetic retinopathy, and two patients (20%) had peripheral neuropathy.

Biochemical-immunological characteristics

The biochemical-immunological characteristics of the ten LADA patients are tabulated in Table 2. All patients had uncontrolled DM. The median fasting postprandial blood sugar and HbA1C were 254.5 ± 130.6 mg/dL, 425 ± 107.8, and 10.7 ± 2, respectively. All patients had hypertriglyceridemia with a median serum triglyceride level of 299 ± 65.8 mg/dL. Nine out of ten patients (90%) had proteinuria defined as 24-hour urinary protein ≥ 150 mg/dL, with a median 24-hour urinary protein of 366 ± 234.2 mg/dL. Median serum creatinine and estimated glomerular filtration rate (e-GFR) were 0.9 ± 0.17 mg/dL and 69.55 ± 22.7 mL/min/1.73 m², respectively. All the patients were positive for the presence of GAD antibodies, with a median anti-GAD antibody level of 25 U/ml (ranging from 12 to >2000 U/ml). The median fasting C-peptide level was 0.23 ± 0.4 μg/ml (range: 0.005 to 1.07 μg/ml).

**Table 2:** Biochemical and immunological characteristics of LADA patients e-GFR: estimated glomerular filtration rate; FBS: fasting blood sugar; GAD: glutamic acid decarboxylase; HbA1C: glycated hemoglobin; LDL: low-density lipoproteins; PPBS: postprandial blood sugar; LADA: latent autoimmune diabetes in adults The variables are expressed as median ± standard deviation.

Case No	FBS (mg%)	PPBS (mg%)	HbA1c	C-peptide (ng/mL)	GAD antibody titre (IU/mL)	Triglycerides (mg/dL)	LDL (mg/Dl)	24-hour urinary protein (mg/dL)	Sr creatinine	e-GFR mL/min/1.73m^2^
1	207	323	8	0.3	17	200	140	150	0.8	112
2	575	657	10.9	0.05	19	373	156	400	1.2	63.6
3	240	360	15.1	0.005	73.22	365	173	302	0.9	97.2
4	171	247	10.3	0.93	12	245	140	356	1.1	70.6
5	375	425	10.6	0.05	445	301	128	50	0.9	66.3
6	368	480	9.8	0.15	>2000	300	156	878	0.9	68.3
7	234	425	12.5	0.63	22	298	178	522	1.2	68.5
8	147	400	8.2	0.8	28	207	135	366	0.7	127.6
9	400	425	11	0.16	>2000	290	114	627	0.8	76.6
10	269	458	5.9	1.07	62.9	388	142	366	1	63.9
Median ± SD	254.5 ± 130.6	425 ± 107.8	10.7 ± 2	0.23 ± 0.4	25 ± 147	299 ± 65.8	141 ± 19.7	366 ± 234.2	0.9 ± 0.17	69.55 ± 22.7

## Discussion

In 1977, Irvine et al. described that 11% of T2DM patients have antibodies against the insulin-producing beta cells, which is a characteristic feature of T1DM, suggestive of an autoimmune pathogenesis [10]. In 1993, Tuomi et al. introduced the term "LADA" to describe the subgroup of patients that share immunological features with T1DM and phenotypical features of T2DM [11]. In 2005, the Diabetes Immunology Society established three criteria for the diagnosis of LADA [6]: onset of the disease after the age of 30 years, presence of any antibody against the islets, and insulin independence for at least six months after diagnosis. In 2006, Fourlanos et al. described five clinical characteristics that were more common in LADA: symptom onset below 50 years of age; presentation with acute symptoms; BMI ≤ 25 kg/m²; history of autoimmune disease in the index case; history of autoimmune disease in the family members. The presence of at least two of these characteristics showed a sensitivity of 90% and a specificity of 71% for diagnosing LADA. A negative predictive value of 99% for a score ≤ one [12]. In our study, the diagnosis of LADA can be made based on both criteria. Considering the Diabetes Immunology Society criteria, all the patients were above 30 years of age at the time of diagnosis, everyone had an elevated titer of GAD antibody, and none needed insulin in the first six months of illness. Considering the Fourlanos criteria, all patients were under 50 years of age at the onset of illness. All patients had a BMI below 25 kg/m². Four patients reported autoimmunity.

Differentiation of LADA patients from T1DM may be challenging, as these patients present with ketoacidosis, are treated with insulin at presentation, have low BMI, and have positive titers for at least one autoantibody. Around 40% of our patients presented with DKA. Patients with maturity-onset diabetes of the young (MODY) may be differentiated from LADA based on a strong family history of DM, residual serum C-peptide levels, and no humoral and cellular immunity to islet cell antigens. The age at onset of LADA sometimes makes it difficult to differentiate it from T2DM, as these patients respond transiently over a period of six months to lifestyle modifications and oral hypoglycemic agents but later cease to respond as β-cell function declines [4]. Those who are misdiagnosed as having T2DM may be treated with oral hypoglycemic drugs and lifestyle modifications instead of being treated with insulin. That results in unwarranted exposure of patients to potential adverse effects of ineffective drugs, persistent hyperglycemia, and ultimately an increased risk of long-term microvascular and macrovascular complications. There was a median delay of 5.5 ± 4.4 years from the time of initial presentation to diagnosis as LADA, exposing our patients to macro- and microvascular complications of DM. 

Buzzetti R et al. found that it is uncommon to see chronic complications at the time of LADA diagnosis [13]. In our patients, despite having an average of 5.5 years of poor glycemic control, all patients had an estimated glomerular filtration rate of 60 ml/min/1.73 m² or higher at presentation. However, one patient had a combination of moderate non-proliferative diabetic retinopathy and peripheral neuropathy. In addition, four patients had mild non-proliferative diabetic retinopathy, and two had peripheral neuropathy. In 2022, Manisha AM et al., in a long-term follow-up study of 186 patients, showed that there was no difference in the incidence of retinopathy and neuropathy among LADA and T2DM patients. However, nephropathy was seen in 25% of LADA patients as compared to 11.5% of T2DM, though it was not statistically significant. Diabetic foot was more common in T2DM as compared to LADA (12.5% vs. 33.1%) [8]. During follow-up, microvascular complications were 25% more common in patients with LADA as compared to patients with T2DM, likely due to less effective glycemic control [14].

## Conclusions

LADA presents serious challenges in its diagnosis and treatment. Identifying specific clinical characteristics, early diagnosis, and appropriate treatment of LADA may prevent further damage to the beta islet cells as well as short-term (like DKA) and long-term microvascular and macrovascular complications of diabetes. Immunological screening in young, thin-built adult diabetics should be considered early in their disease course. Timely adoption of insulin therapy also appears to benefit these patients. Further studies with larger sample sizes are needed to better define the socioeconomic burden and long-term outcomes of LADA.
